# ID 86 - Características e Estrutura de Modelos de Avaliação Econômica em Hemofilia: um estudo metaepidemiológico

**DOI:** 10.5327/2237-9622.2025.v34s1.86

**Published:** 2025-11-25

**Authors:** Bruna Marmett, Gilson Dorneles, Nayê Schneider, Celina Migliavaca, Maicon Falavigna

**Keywords:** hemofilia, avaliação econômica em saúde, modelagem, profilaxia

## Abstract

**Introdução:**

A hemofilia é uma desordem hemorrágica rara caracterizada por deficiências nos fatores de coagulação com significante impacto na morbidade e na qualidade de vida dos pacientes.

A hemofilia A está relacionada à deficiência do fator VIII, enquanto a hemofilia B é decorrente da deficiência do fator IX. Um dos desafios terapêuticos reside no surgimento de autoanticorpos (inibidores) contra os fatores de coagulação, comprometendo a eficácia do tratamento convencional.

Dada a complexidade da doença e suas alternativas de tratamento, faz- se necessário o entendimento de modelos de avaliação econômica em saúde (AES) de alternativas terapêuticas no tratamento da hemofilia. O objetivo deste trabalho foi descrever as características metodológicas de estudos de avaliação econômica para a avaliação de terapias para o tratamento de hemofilias

**Método:**

Foi realizada uma abordagem metaepidemiológica por meio de base de dados (MEDLINE/PubMed) e websites de importantes agências de saúde nacionais (Conitec) e internacionais (NICE, CADTH e ICER). Foram considerados elegíveis publicações científicas e dossiês que conduziram avaliação econômica completa publicadas nas línguas portuguesa ou inglesa a partir de 2018 até dezembro de 2023. A apresentação dos resultados para sumarizar as informações obtidas dos documentos ocorreu por análise descritiva.

**Resultados:**

Quarenta e uma publicações foram consideradas elegíveis, sendo identificado maior número de estudos de avaliação econômica para hemofilia A (85,7%). Tecnologias do tipo imunobiológicos (38,1%) e FVIII recombinante (33,3%) foram as intervenções mais frequentes. Em 50% das submissões foram realizadas análises de custo-utilidade, enquanto 33,34% relataram análise de custo-efetividade e 16,66% adotaram análise de custo-minimização; sendo que anos de vida ajustados pela qualidade foi o principal desfecho em saúde (69%), seguido por eventos de sangramento tratados (44,4%) e sangramentos em articulações tratados (16,6%). Os principais modelos adotados foram o de Markov (75%) e árvores de decisão (10%). Os custos médicos mais frequentes foram relacionados à administração do tratamento (29,6%), aquisição do tratamento (29,6%), monitoramento da doença (11,0%), manejo de sangramentos (24,2%), cirurgia (16,8%), hospitalização (15,7%), eventos adversos do tratamento (14,7%) e tratamento de artropatia (8,4%). Os estudos de custo-utilidade consideraram sobretudo o grau de sangramento (47,7%), cirurgia (15,9%) e artropatia (15,9%) como principais eventos de saúde com impacto sobre os dados de utilidade de pacientes com hemofilia. A desutilidade por evento adverso foi mensurada em 29%. As análises de sensibilidade determinística e probabilística foram realizadas em 70% e 60% das publicações avaliadas, respectivamente.

**Conclusão:**

Diversos novos tratamentos vêm sendo propostos para o tratamento de hemofilias, desde fatores de coagulação recombinantes a terapias gênicas, o que demandarão estudos avaliando a custo-efetividade dessas tecnologias. Identificamos uma ampla variação nos tipos e nas estruturas das análises econômicas utilizadas para a avaliação de terapias em hemofilia. Essa heterogeneidade observada na literatura sinaliza a impossibilidade de uso de um modelo-padrão a ser adaptado para novas análises econômicas, devendo cada tecnologias ser adequadamente contextualizada dentro do sistema de saúde-alvo, de forma a avaliar a melhor forma de prover subsídios para a tomada de decisão.

